# Supplementary material for: Associations of the TyG index with albuminuria and chronic kidney disease in patients with type 2 diabetes
Source: PLoS One. 2024 Oct 28;19(10):e0312374. doi: 10.1371/journal.pone.0312374 (PMC11515963; doi:10.1371/journal.pone.0312374)
Supplement: S2 File — (PDF) [file pone.0312374.s003.pdf]

editage

# Editing Certificate

This document certifies that the manuscript listed below has been edited to ensure language and grammar accuracy and is error free in these aspects. The edit was performed by professional editors at Editage, a brand of Cactus Communications. The author's core research ideas were not altered in any way during the editing process. The quality of the edit has been guaranteed, with the assumption that our suggested changes have been accepted and the text has not been further altered without the knowledge of our editors.

MANUSCRIPT TITLE

**Associations of the TyG index with albuminuria and chronic kidney disease in patients with type 2 diabetes**

AUTHORS

**Xiaonan Li, Yuehui Wang**

ISSUED ON

**May 25, 2024**

JOB CODE

**ZMUOP_2**

THE EDITAGE Promise QUALITY GUARANTEE

Prabh Grewal
Senior Vice President - Editage

editage helping you get published

Since 2002, Editage has helped over 430,000 authors publish around 1.2 million research papers in scholarly journals across over 1000 disciplines through editorial, translation, transcription, and publication support services. Editage is a brand of Cactus Communications (cactusglobal.com), a science communication and technology company.

CACTUS.

GLOBAL :
+1(833) 979-0061 | request@editage.com

CHINA :
400-120-3020或021-6020-9400 | fabiao@editage.cn
