# Supplementary material for: Associations of the TyG index with albuminuria and chronic kidney disease in patients with type 2 diabetes
Source: PLoS One. 2024 Oct 28;19(10):e0312374. doi: 10.1371/journal.pone.0312374 (PMC11515963; doi:10.1371/journal.pone.0312374)

虚拟桌面用户使用说明

1. 通过人口健康数据中心网站（https://www.ncmi.cn/），进入虚拟桌面登录页面。

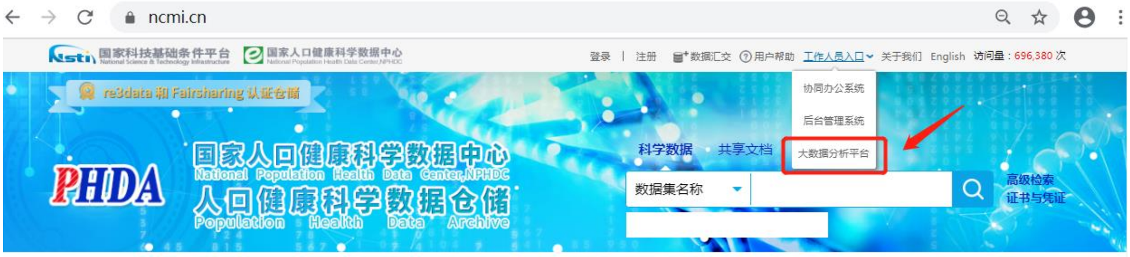

输入数据中心分配给您的用户名和密码。

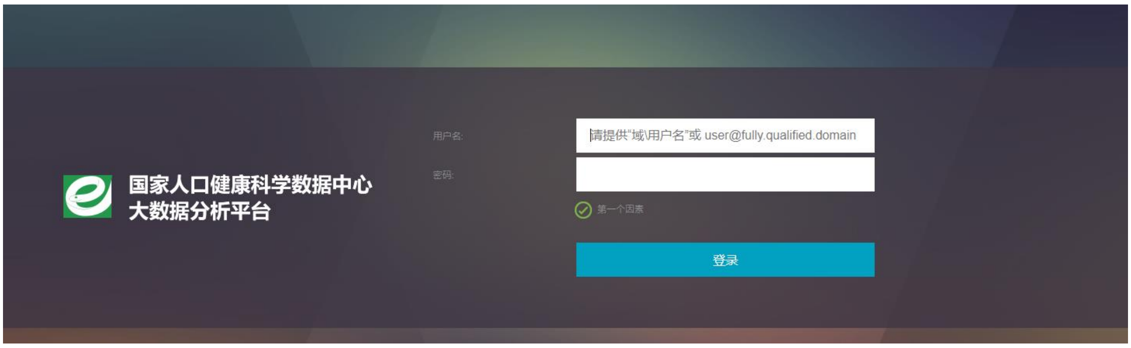

输入动态密码（数据申请时所留手机号的手机短信）

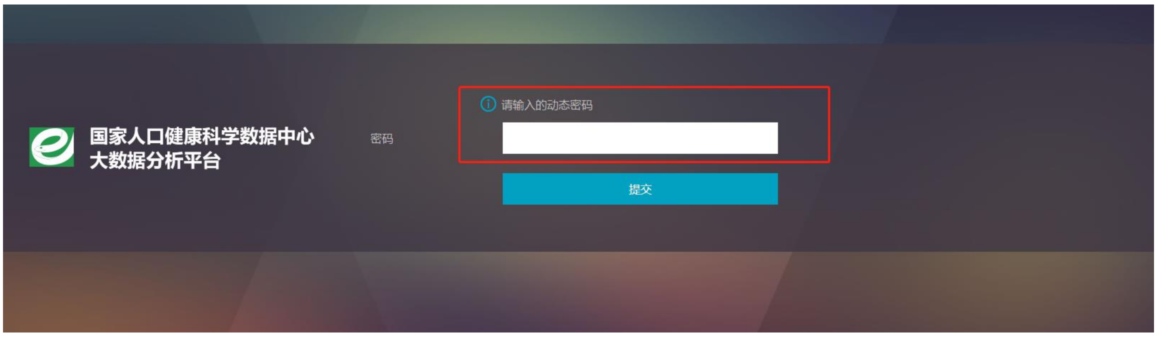

注：动态码 5 分钟有效期，其中对同一个手机号有如下限制：30 秒内发送短信条数不超过 1 条，一个小时内最多发送短信条数不超过 10 条，1 个自然日内发送短信条数不超过 20 条短信）

2. 初次使用时，会检测您的个人电脑是否安装插件，点击“检测 Receiver”。

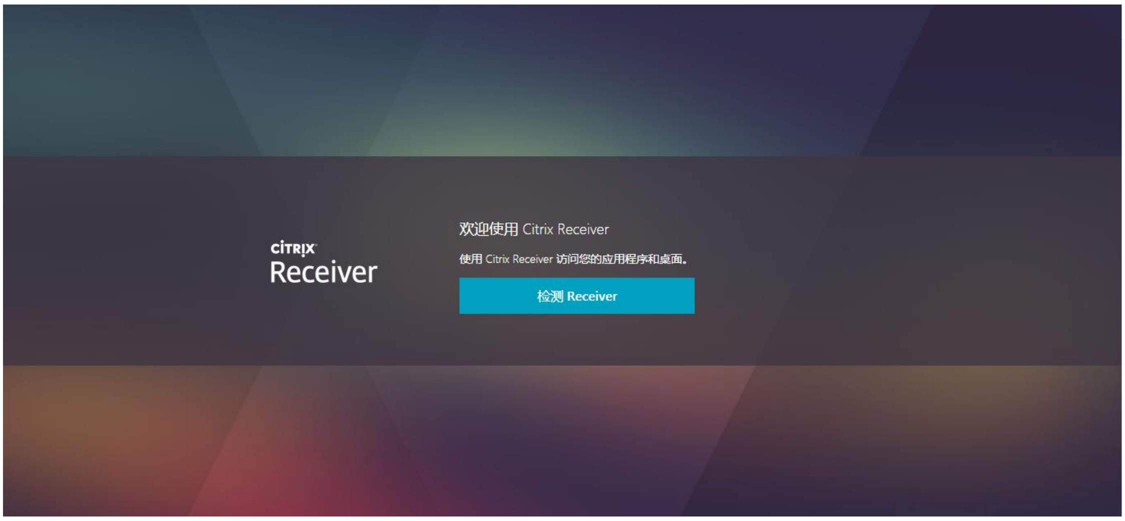

勾选“我同意 Citrix 许可协议”，并点击“下载”，下载客户端。

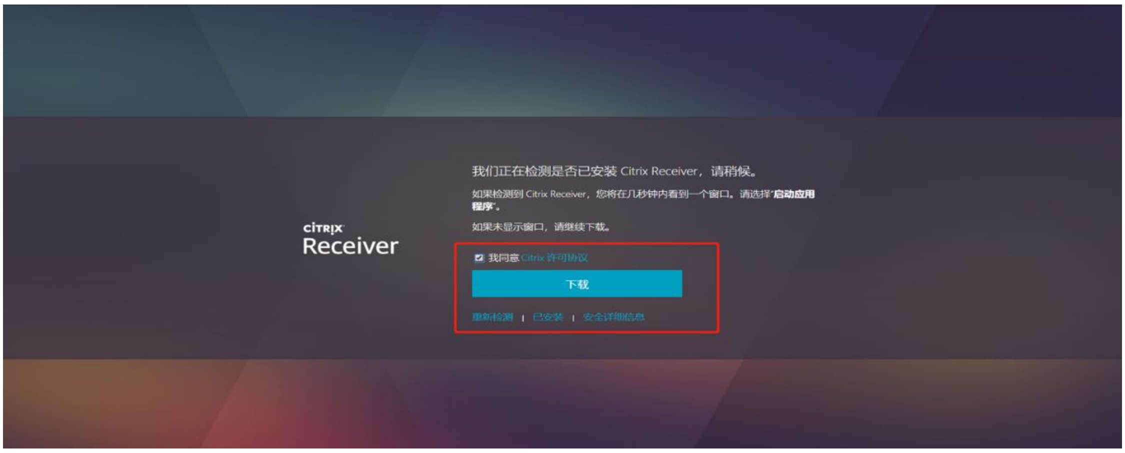

3. 按照默认步骤安装客户端，至完成。

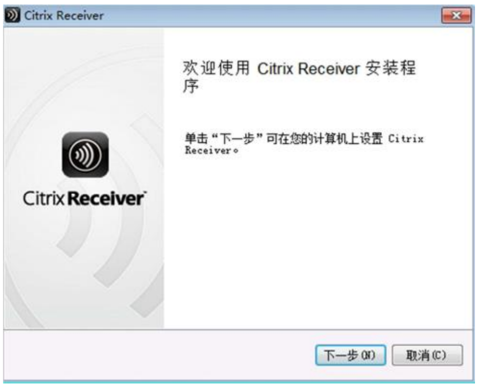

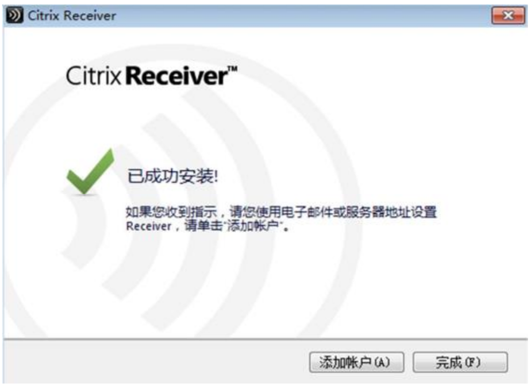

4. 点击“Win10 桌面”图标，进入虚拟桌面。

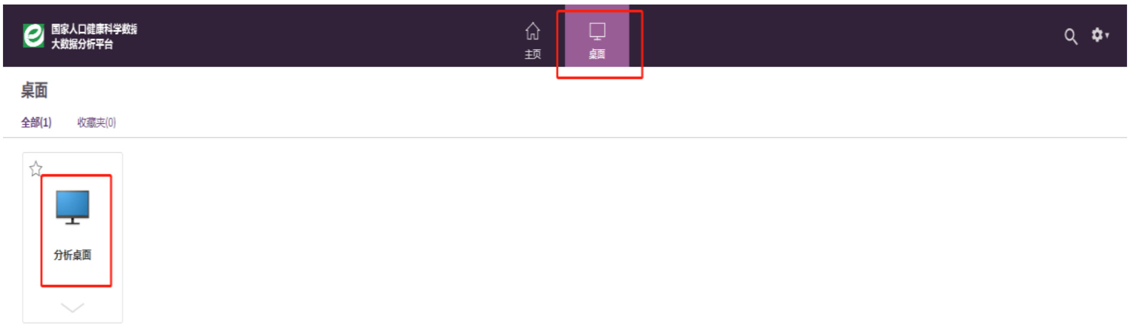

注意：第一次进入虚拟桌面时间较长，请耐心等待。

5. 所有内容均在桌面的“个人文件夹”中，所有操作均在该文件夹内进行，请不要删除该文件夹中的任何内容。

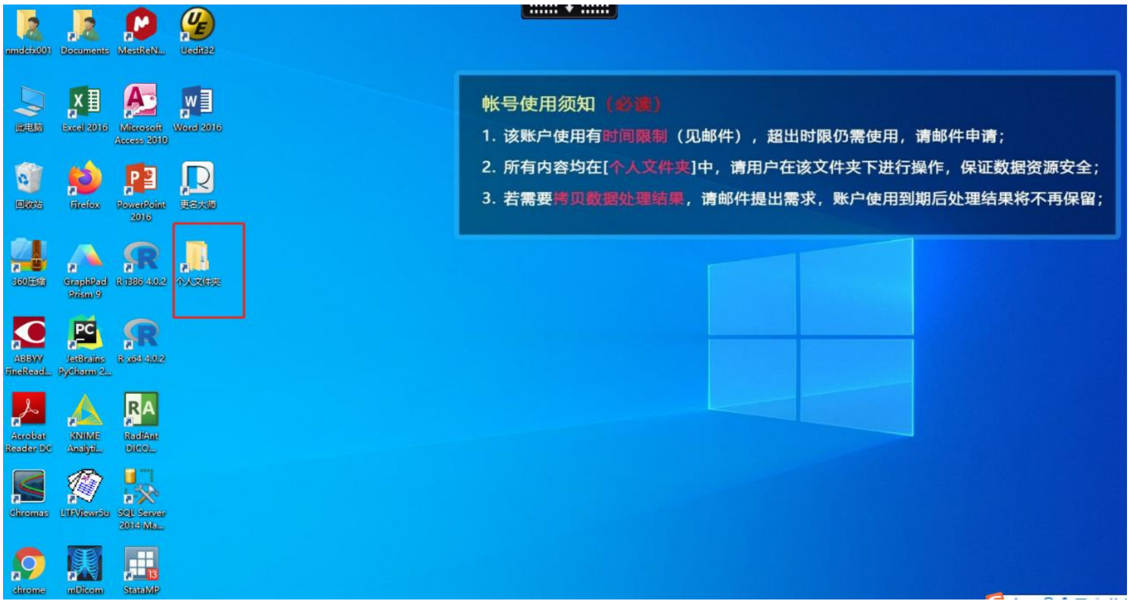

注意：若您在该文件夹中进行“删除文件”的操作将是彻底删除，无法找回，请慎重操作。

6. 完成所有操作之后，如您不再使用虚拟桌面，请关闭虚拟桌面。

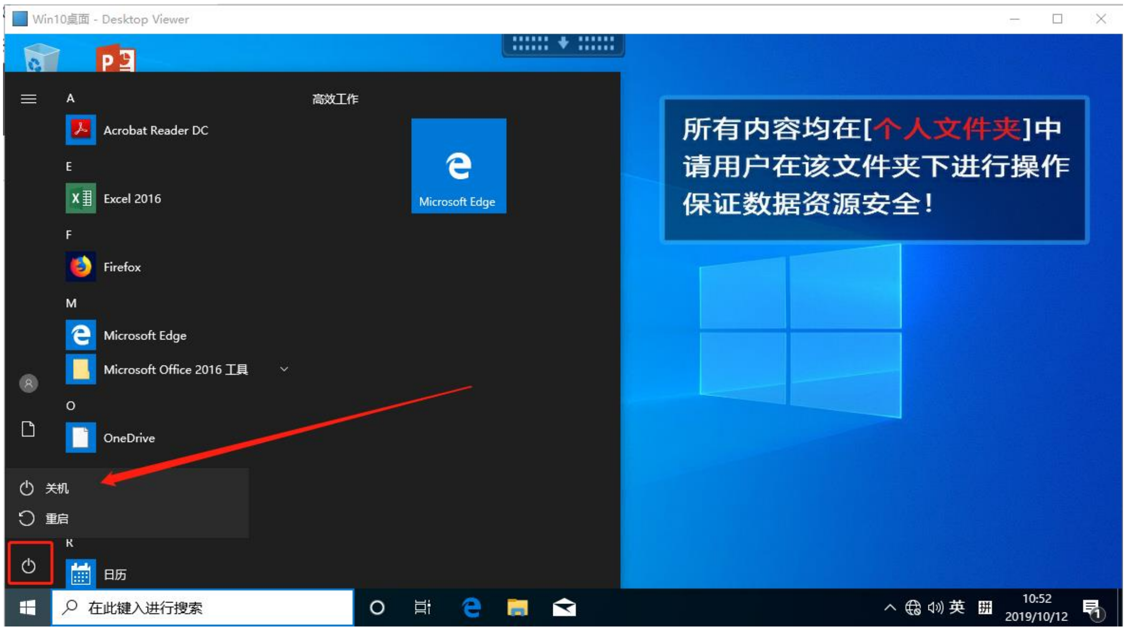

<u>注意：若您的个人电脑为 Win10，任务栏与虚拟桌面相似，不要误操作关闭个人电脑。</u>

7. 使用中可能出现的问题：

   1） 虚拟桌面中进行文件操作可能出现延迟、缓慢等情况，为正常现象，请耐心等待；

   2） 30 分钟不做任何操作，虚拟桌面将断开连接，重新打开即可。

   3） 出现以下页面可直接点击“关闭”

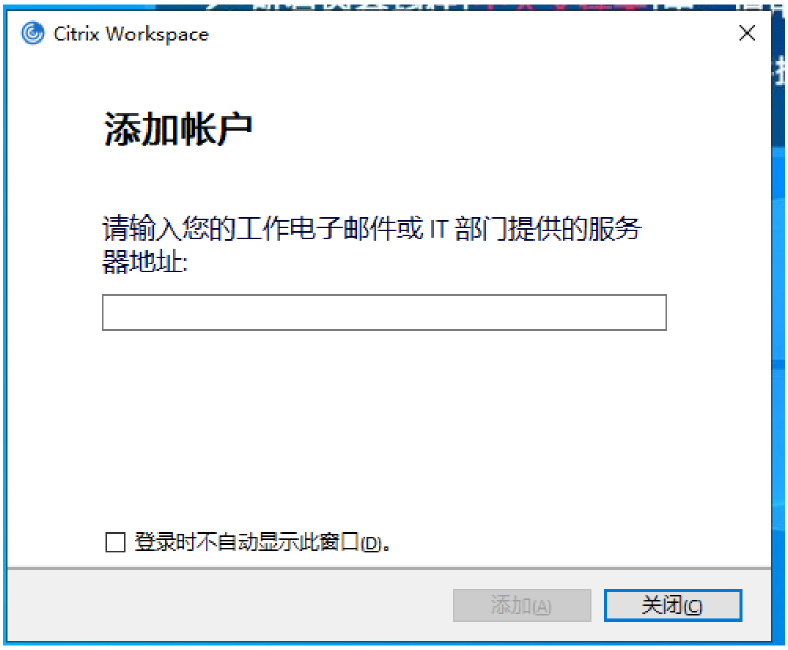

4) 使用 mac 操作系统常见证书问题，如图示，参照 https://jingyan.baidu.com/article/5225f26ba6dbfce6fb090856.html 中说明进行操作；建议使用 windows 操作系统进入虚拟桌面

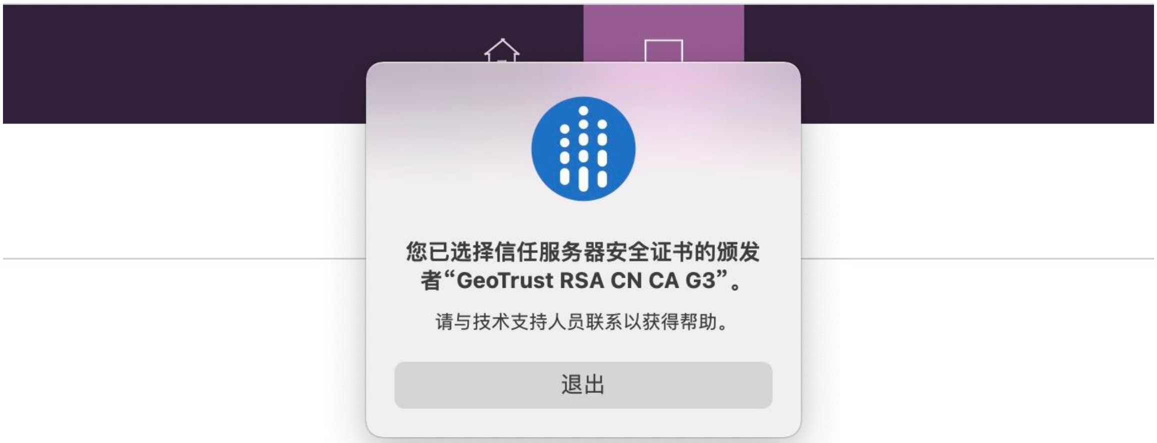

Supplement: S3 File — (PDF) [file pone.0312374.s004.pdf]
