# Supplementary material for: Associations of the TyG index with albuminuria and chronic kidney disease in patients with type 2 diabetes
Source: PLoS One. 2024 Oct 28;19(10):e0312374. doi: 10.1371/journal.pone.0312374 (PMC11515963; doi:10.1371/journal.pone.0312374)
Supplement: Attachment — Submitted filename: Response to Reviewers.docx [file pone.0312374.s005.docx]

**Manuscript ID:** PONE-D-24-07664

**Manuscript title:** Association of TyG index with albuminuria and Chronic Kidney Disease with Type 2 Diabetes Mellitus

Dear Editor:

Thank you very much for your attention, evaluation, and comments on our paper "Association of TyG index with albuminuria and Chronic Kidney Disease with Type 2 Diabetes Mellitus". We have revised the manuscript according to your kind advice and the reviewer’s detailed suggestions and tried our best to revise the manuscript. Please find the responses to the reviewers below. We sincerely hope this manuscript will be finally accepted for publication in "PLOS ONE". We would love to thank

you for allowing us to resubmit a revised copy of the manuscript and we highly appreciate your time and consideration.

The following is the point-by-point response to the editor and reviewers.

Revised portions are marked in purple in the revised paper. The main corrections in the paper and the responses to the editor’s comments are the following

Editor #

**Comment 1**: Please ensure that your manuscript meets PLOS ONE's style requirements, including those for file naming. The PLOS ONE style templates can be found at https://journals.plos.org/plosone/s/file?id=wjVg/PLOSOne_formatting_sample_main_body.pdf and

<https://journals.plos.org/plosone/s/file?id=ba62/PLOSOne_formatting_sample_title_authors_affiliations.pdf.>

**Response**: Thanks to your prompt attention, we have revised the manuscript according to PLOS ONE's style requirements.

**Comment 2**. Thank you for stating the following in your Competing Interests section: “no” Please complete your Competing Interests on the online submission form to state any Competing Interests. If you have no competing interests, please state "The authors have declared that no competing interests exist.", as detailed online in our guide for authors at http://journals.plos.org/plosone/s/submit-now.This information should be included in your cover letter; we will change the online submission form on your behalf.

**Response**: We appreciate your suggestions and we have made the appropriate changes. The statement of “The authors have declared that no competing interests exist” are put in the cover letter and manuscript.

**Comment 3**. For studies involving third-party data, we encourage authors to share any data specific to their analyses that they can legally distribute. PLOS recognizes, however, that authors may be using third-party data they do not have the right to share. When third-party data cannot be publicly shared, authors must provide all information necessary for interested researchers to apply to gain access to the data. (https://journals.plos.org/plosone/s/data-availability#loc-acceptable-data-access-restrictions)For any third-party data that the authors cannot legally distribute, they should include the following information in their Data Availability Statement upon submission: a) A description of the data set and the third-party sources) If applicable, verification of permission to use the data set. c) Confirmation of whether the authors received any special privileges in accessing the data that other researchers would not haved) All necessary contact information others would need to apply to gain access to the data.

**Response**: The data for this study were obtained from the Population Health Data Archive's Diabetes Complications Data Set( A Public database) from 01/2013 to 12/2017. Users can request and access data at https://www.ncmi.cn//phda/dataDetails.do?id=CSTR:A0006.11.A0005.201905.000282, applicants can also get information about the application by contacting the email Icyx301@126.com.

**Comment 4**. PLOS requires an ORCID iD for the corresponseing author in Editorial Manager on papers submitted after December 6th, 2016. Please ensure that you have an ORCID iD and that it is validated in Editorial Manager. To do this, go to ‘Update my Information’ (in the upper left-hand corner of the main menu), and click on the Fetch/Validate link next to the ORCID field. This will take you to the ORCID site and allow you to create a new iD or authenticate a pre-existing iD in Editorial Manager. Please see the following video for instructions on linking an ORCID iD to your Editorial Manager account: <https://www.youtube.com/watch?v=_xcclfuvtxQ>

**Response**: Thanks for the advice, I already have an ORCID iD, and it is validated in Editorial Manager.

**Comment 5**: Please include your full ethics statement in the ‘Methods’ section of your manuscript file. In your statement, please include the full name of the IRB or ethics committee who approved or waived your study, as well as whether or not you obtained informed written or verbal consent. If consent was waived for your study, please include this information in your statement as well.

**Response**: We appreciate your advice, the study was conducted from a third-party database with identifiable data, and the data on Diabetes Complications Data Set were stored in the Population Health Data Archive, which contains 87 variables for 3,000 patients with T2DM. A secondary analysis of the data from this data set was performed. The study was a retrospective study and conducted using a third-party database with identifiable data; and the research project did not involve personal or commercial interests, the requirement of ethical approval for this was waived by the Institutional Review Board of the First Hospital of Jilin University.The need for written informed consent was waived by the Institutional Review Board of the First Hospital of Jilin University due to retrospective nature of the study.

Revised portions are marked in red in the revised paper. The main corrections in the paper and the response to the reviewer’s comments are as follows

Reviewer #1

**Comment 1** This manuscript examined the connection between the triglyceride-glucose (TyG) index, albuminuria, and chronic kidney disease (CKD) in individuals with type 2 diabetes mellitus (T2DM). They analyzed data from 3000 patients, of whom 1048 met the study's criteria. CKD was determined by a glomerular filtration rate (eGFR) < 60 ml/min-1.73 m-2 or a urinary albumin-to-creatinine ratio (UACR) ≥ 30 mg/g, while albuminuria was defined as UACR ≥ 30 mg/g. Results indicated a positive association between the TyG index and both albuminuria and CKD, with higher odds ratios observed for those in the highest TyG index quartile. the whole manuscript sections (introduction, method, result, discussion) should be revised. The manuscript does not follow a standard structure of manuscript writing. I recommend a native English-speaking revision to address the many stylistic issues, the manuscript lacks fluency and the English in the text is EXTREMELY poor, complete revision is necessary. these are some examples of the text that should be revised.

**Response**: Thank you very much for your suggestions and your kind work. I'm very sorry for the poor English, I've invited a native English speaker to revise the whole manuscript, and we've also revised the article point by point according to your comments on the problems you've raised in the article, so I hope our revisions can make your reading more satisfying.

**Abstract**

**Comment 2**: The objective should be explained comprehensively, pointing to the aim, benefits, and necessity of the study.

Response: Thank you for your serious work, We've taken your suggestion and written it up. As the incidence of diabetes mellitus increases, diabetes mellitus-related kidney injury also shows a trend of gradual increase, there is no targeted treatment for diabetic kidney injury, so the prevention is greater than the treatment. Insulin resistance is an important risk factor for diabetic kidney injury, and the TyG index is economical, cheap, and easy to promote the resistance of insulin as an indicator, and it is less explored in the diabetic kidney injury research. Therefore, the purpose of this study is to investigate the relationship between the TyG index and albumin and CKD of T2DM and to search for an excellent index that can provide clinicians with an early evaluation of the disease. You can see it below

Objective: Diabetes-related kidney disease reduces patients' quality of life, increases the risk of death, and is associated with insulin resistance (IR). The triglyceride-glucose (TyG) index is a simple and inexpensive alternative to IR measurement. Furthermore, the relationship between albuminuria and chronic kidney disease (CKD) in type 2 diabetes mellitus (T2DM) remains unclear. Therefore, we aimed to investigate the association of TyG index with albuminuria and CKD in patients with T2DM.

**Comment 3:** In the method section, mention the timeline of data acquisition.

**Response**: Thank you for your suggestion. the timeline of data acquisition is from 01/2013 to 12/2017., and we have also added a flowchart (Fig 1) of participant inclusion in the study to more clearly define our process.

**Comment 4**: In the result section, include patient characteristics such as mean age, sex, BMI, etc.

**Fig 1. Flowchart for inclusion of populations**

**Response**: Thanks for your suggestion, I have described the characteristics of the inclusion population in the results section. The changes to the results section can be seen in the revised text and below.

Results: We identified 1048 subjects, 63.03% of whom were men. The mean age was 46.21 years, and the mean body mass index was 26.742 kg/m2. CKD and albuminuria detection rates showed an increasing trend in the different TyG subgroups. (p = 0.008, p = 0.006). Using the Q1 group as a baseline, the risk of albuminuria and CKD was significantly greater in the group Q3 (OR = 1.514, 95% CI 1.121-2.047 P = 0.05), and the same result was obtained after adjusting for covariates (OR = 2.241, 95% CI 1.245-4.034, P = 0.007). Subgroup analyses revealed a significant increase in the incidence of albuminuria and CKD in the group Q3 compared to that in the Q1 group.

**Comment 5**: In the conclusion, clarify the clinical usage of these findings

**Response**: We are very grateful to the reviewers for your comments, our findings have clinical and public health significance because of the increasing prevalence of diabetes and the growing population with diabetes-related kidney injury and the TyG index is positively associated with albuminuria and CKD in patients with T2DM and may be a marker for predicting the occurrence of early kidney injury in patients with T2DM. So clinicians can perform early testing TyG index to identify early kidney damage and intervene aggressively to reduce proteinuria and CKD and improve prognosis, thereby reducing the risk of chronic complications of diabetes mellitus. we have changed the results section based on your suggestion. We have revised the abstract, the exact description of which can be seen in the revised manuscript and below

Objective: Diabetes-related kidney disease reduces patients' quality of life, increases the risk of death, and is associated with insulin resistance (IR). The triglyceride-glucose (TyG) index is a simple and inexpensive alternative to IR measurement. Furthermore, the relationship between albuminuria and chronic kidney disease (CKD) in type 2 diabetes mellitus (T2DM) remains unclear. Therefore, we aimed to investigate the association of TyG index with albuminuria and CKD in patients with T2DM.

Methods: Data from 01/2013-12/2017 period were obtained from the Population Health Data Archive's Diabetes Complications Data Set. A total of 1048 patients with T2DM were included in this study. CKD is defined as an estimated glomerular filtration rate < 60 ml/min^-1^.1.73 m^-2^ or a urinary albumin-to-creatinine ratio (UACR) ≥ 30 mg/g. Albuminuria is defined as a UACR ≥ 30 mg/g. The TyG index is calculated by measuring the triglyceride and fasting blood glucose levels. Logistic regression models were used to analyze the association between albuminuria, CKD with T2DM and TyG index.

Results: We identified 1048 subjects, 63.03% of whom were men. The mean age was 46.21 years, and the mean body mass index was 26.742 kg/m^2^. CKD and albuminuria detection rates showed an increasing trend in the different TyG subgroups. (*p* = 0.008, *p* = 0.006). Using the Q1 group as a baseline, the risk of albuminuria and CKD was significantly greater in the group Q3 (OR = 1.514, 95% CI 1.121-2.047 *P* = 0.05), and the same result was obtained after adjusting for covariates (OR = 2.241, 95% CI 1.245-4.034, *P* = 0.007). Subgroup analyses revealed a significant increase in the incidence of albuminuria and CKD in the group Q3 compared to that in the Q1 group.

Conclusions: The TyG index is positively associated with albuminuria and CKD in patients with T2DM and may be a marker for predicting the occurrence of early kidney injury in patients with T2DM. Clinicians should test this indicator early to detect lesions and improve patient prognosis.

**Comment 5** The whole manuscript sections (introduction, method, result, discussion) should be revised. The manuscript does not follow a standard structure of manuscript writing. I recommend a native English-speaking revision to address the many stylistic issues, the manuscript lacks fluency and the English in the text is EXTREMELY poor, complete revision is necessary. these are some examples of the text that should be revised:

Response: Thank you very much for your suggestions, we have carefully revised the objectives, methods, results, and conclusions of the abstract according to your suggestions, and we hope that our revisions will make you have a satisfactory reading experience!

**Introduction**

**Comment 6** The results concerning hard end-points should be stated. In my opinion, it is too brief and not focused on clinical aspects clarifying the specific factors, tests, or markers evaluated and their significance in understanding the pathological roles in patients with CKD. These are some of the many grammatical, stylistic, and spelling errors: “Chronic kidney disease (CKD) is a microvascular complication of diabetes mellitus (DM) (1). As the prevalence of CKD increases, so does the risk of cardiovascular disease, end-stage renal disease (ESRD), and cumulative mortality (2)” The whole paragraph should be revised.

**Response**: Thank you for your suggestion, we have rewritten the introduction section and have invited native English speakers to correct the grammatical, stylistic, and spelling errors. I have enriched the introduction with a description of the Tyg index to the clinic, which includes specific factors for its assessment and specific applications and explains the significance of the Tyg index in terms of its pathological role in patients with CKD. You can see the overall changes to the introduction in the revised manuscript and in the response to **comment 9**

**Comment 7**: Paraphrase "CKD WAS defined as estimated glomerular filtration …" to "CKD is defined as estimated glomerular filtration…"

**Response**: Thank you for your suggestion, we have changed it to “CKD is defined as estimated glomerular filtration…"

**Comment 8**: some well-defined and basic scientific facts are mentioned as scientific suggestions for example: “It has been suggested that insulin resistance (IR) is closely associated with an increased risk of kidney disease in diabetic populations”

**Response**: Thank you for your suggestion, we have rewritten this section and avoided the phenomenon you mentioned.

**Comment 9**: There should be an explanation regarding the definition of the TyG index, its utility, and previous research on this index. Delve into the concept of clinical importance and the superiority of using this index in CKD. Overall, significant revisions are required for structural, grammatical, and clarity issues. Additionally, the clinical implications of the findings need further discussion.

**Response**: Thank you for your suggestions, I have revised the introduction section as a whole based on your comments. The TyG index is a combination of FPG and TG. The specific formula is ln [FPG (mg/dl) *TG (mg/dl)], we discuss the previous research on this index and also describe its superiority and reliability compared to HOMA-IR and HIEC tests for renal disease, and discuss and summarize the clinical importance of its use.The overall introduction to introduction can be seen in the revised manuscript marker red and below.

Introduction

Type 2 diabetes mellitus (T2DM) is the most common metabolic disorder that leads to numerous microvascular and macrovascular complications (1). Chronic kidney disease (CKD), a major microvascular complication of T2DM, affects nearly half of the patients with T2DM, and approximately 30% of these patients eventually progress to end-stage renal disease (ESRD) (2-5). CKD is defined as an estimated glomerular filtration rate (eGFR) < 60 ml/min^-1^.1.73 m^-2^ or increased levels of urinary albumin-to-creatinine ratio (UACR ≥ 30 mg/g) (6, 7). T2DM is a major cause of progression to end-stage renal disease (ESRD). Diabetes-related ESRD severely affects the quality of life and treatment of patients, imposing a significant economic burden (8). Therefore, delaying progression to ESRD is a major challenge in the management of patients with T2DM.

Insulin resistance (IR), which is characterized by the inability of cells to respond to the action of insulin, is a prominent feature of T2DM and is significantly associated with various diseases, including cardiovascular disease, CKD, and cognitive impairment (9-11). Studies have shown that IR may be related to increased glomerular hydrostatic pressure, resulting in increased renal vascular permeability, eventually leading to glomerular hyperfiltration (8). Another hypothesis is that metabolic changes associated with IR cause glomerulosclerosis, glomerular hypertrophy, tubulointerstitial inflammation, and fibrosis, ultimately leading to albuminuria and accelerated CKD progression (12, 13). Therefore, assessing the IR is crucial for understanding the occurrence and progression of T2DM-related kidney diseases.

Studies have indicated that the TyG index, calculated by combining fasting glucose and triglycerides, has a strong correlation with IR, as evaluated by the Homeostatic Model Assessment for Insulin Resistance (HOMA-IR) and the Hyperinsulinemic-Euglycemic Clamp (HIEC) test (14-16). Unlike the traditional IR assessment methods (HOMA-IR and HIEC), the TyG index is cost-effective and readily accessible. Multiple studies have demonstrated that the performance of the TyG index in the assessment of IR is consistent with or superior to that of HOMA-IR(17-19). Thus, the TyG index is emerging as a novel and promising alternative marker for insulin resistance.

Numerous studies have demonstrated a close relationship between the TyG index, T2DM, and vascular lesions (20-24). Wang L et al. reported that the TyG index, which is independent of known cardiovascular risk factors, can serve as an independent risk factor for predicting the occurrence of major adverse cardiovascular events in patients with T2DM (22). Moreover, IR has been consistently associated with increased urinary albumin excretion. A cross-sectional study involving 9,872 participants revealed that the TyG index was closely related to albuminuria in the American population (25). A large observational population-based cohort study conducted by Fritz et al. revealed that the TyG index was independently associated with an increased risk of end-stage renal disease (ESRD), and nearly half of the association between BMI and ESRD risk of ESRD is mediated by the TyG index (26). Similarly, in a recent cohort study on middle-aged white men conducted by KUNUTSOR S K et al., a higher TyG index was associated with an increased risk of CKD (27). However, there are few studies on the relationship between the TyG index, albuminuria, and CKD in individuals with T2DM. Therefore, in patients with a high incidence of T2DM, CKD, and albuminuria, clinicians should perform early TyG assessments to detect early kidney damage so that proactive interventions can be performed. This may reduce the incidence of chronic kidney disease and albuminuria, improve the prognosis, and minimize the risk of chronic complications associated with diabetes. Therefore, we aimed to analyze the relationship between the TyG index and the risk of albuminuria and CKD in patients with T2DM.

Methods:

**Comment 10** This section does not follow the standard structure of a manuscript and should be paraphrased. For example, is written in multiple title sections for each item which can be written in a paragraph. Consider replacing methodology with methods.

**Response:** Thank you for your suggestion, we have already merged some of the items that could be merged applied a paragraph to the description, and deleted the methodology item.

Results

**Comment 11** The whole section should be revised. In the first paragraph of the results section there should be a brief presentation of the patients' characteristics (mean age, sex, BMI, etc.)

**Response**: Thanks for your advice. We have briefly described the patient's characteristics，the specific changes you can see below

A total of 1048 participants met the inclusion criteria and were finalized for analysis. The mean age was 46.21 years, the mean body mass index (BMI) was 26.742 kg/ m2, and 63.03％ were men. Baseline participants' characteristics stratified by the TyG index are shown in Table 1. Compared with group Q1, group Q3 has a higher proportion of albuminuria, DKD, poor glycemic control retinopathy, hyperuricemia, and fatty liver (*P* < 0.05). Significant statistical differences were observed in the levels of FPG, HBA1c, albumin, TG, TC, HDL, BUN, creatinine, UA, GGT, and ALP among the three groups (*P* < 0.05). Pairwise comparisons between Group Q3 and Groups Q1 and Q2 showed statistically significant differences in FPG and HBA1c.

**Comment 12:** point to the main findings and after that consider mentioning the tables.

**Response**: Many thanks for your suggestion, we have pointed to the main findings, and after that consider mentioning the tables

Discussion:

**Comment 13**: Aside from many scientific errors, this section does not follow the standard structure of a manuscript and should be paraphrased. The topics discussed in the discussion have not been presented in a relevant, orderly, and coherent manner.

**Response**: We appreciate your comments and apologize for the problems with our discussion section, we have revised the manuscript according to your comments in detail, you can see it in the revised manuscript marked in red and as follows.

Discussions

This study investigated the potential correlation between TyG index and the presence of albuminuria and CKD in patients with T2DM. In this single-center retrospective cohort study, we found a strong positive correlation between the TyG index, albuminuria, and CKD with T2DM. After adjusting for multiple covariates, the positive correlation remained stable, suggesting that baseline TyG index is an independent predictor of albuminuria and CKD in patients with T2DM. This association remained consistent across subgroups of sex, age, HbA1c level, hypertension, fatty liver, and hyperuricemia.

IR, an important feature of T2DM, is particularly associated with nephropathy and a decline in kidney function and is present in the early stages of CKD (28-30). Albuminuria, a marker of early kidney damage, is closely associated with IR (31, 32). PAN Y et al. reported that patients with an elevated TyG index have a greater risk of developing albuminuria, which is consistent with our results and is more pronounced in patients with poor glycemic control (33). There are several possible mechanisms through which IR may be associated with kidney damage. The kidney contains a variety of insulin-sensitive cells that express insulin receptors (34). When IR occurs, insulin signaling is impaired and glomerular filtration pressure is increased, leading to glomerular hyperfiltration (35), cytoskeletal rearrangement (36), mitochondrial dysfunction (37) and inflammation (34), resulting in renal hemodynamics, disruption of podocyte viability, and accelerated progression of CKD by facilitating glomerular capillary dilation and tubular fibrosis (12, 13) Therefore, early detection of IR is crucial for the prevention of kidney disease.

Currently, the gold standard for assessing IR is the HIEC test; however, it is invasive and expensive, used only for academic research, and not applied in clinical practice (38). The widely used HOMA-IR index, which assesses β-cell function and insulin resistance, has limited value in patients undergoing insulin therapy or those without functional β-cells, and serum insulin measurement is not widely used in clinical practice (38). The TyG index, calculated using FPG and TG levels, assesses IR in both diabetic and non-diabetic individuals and is equivalent to or even better than HOMA-IR (14-16, 39). This alternative method is simple, convenient, and low-cost, does not require insulin quantification, and can be used in all patients regardless of their insulin treatment status (40). Therefore, in our study, we adopted the TyG index as an alternative biomarker for IR because it is readily accessible in routine clinical practice and widely used.

The TyG index, a new surrogate for insulin resistance measurement, is increasingly supported by evidence of its important role in predicting vascular diseases (41, 42) A meta-analysis indicated that the TyG index is positively associated with the incidence rates of heart failure and adverse outcomes. Similarly, a study also suggests that the prevalence of other common cardiovascular and cerebrovascular diseases, including arteriosclerosis, coronary artery calcification, coronary artery disease, myocardial infarction, and atrial fibrillation, is significantly related to a high TyG index (43-48). However, the association between the TyG index and renal microvascular damage was low. Our study showed that an elevated TyG index is significantly associated with the risk of albumin deficiency and CKD in patients with T2DM. A high TyG index has been shown to exacerbate renal dysfunction by promoting insulin resistance and related metabolic disturbances (49), and it is a reliable and convenient prognostic marker for adverse outcomes in patients with kidney disease (50). A cohort study based on CHARLS data revealed an increased risk of CKD in the highest quartile compared to that in the lowest quartile (51), which is the same as that found in our study. Interestingly, Vinas et al. conducted basic experiments to demonstrate that female mice were more resistant to kidney injury than male mice (52). Saliba et al. reported the same result in humans, which may be related to the estrogen-AMPK pathway (53). However, in our study, we did not find any difference in albumin and CKD levels by sex, even after adjusting for risk factors and sex for stratification; this may be related to the ethnicity of the included population, geographic region, and the number of patients included, which needs to be further explored.

Our study had several limitations. First, it was retrospective, precluding the establishment of a causal relationship between our variables of interest and outcomes, and there may have been reporting and selection biases. Second, although potential confounders were controlled as covariates in the multivariate regression model, it is important to acknowledge that the impact of unrecorded confounders cannot be categorically excluded. Finally, our study was a single-center study with a moderate sample size; future research should include large-scale multicenter longitudinal and prospective studies to further investigate the effects and mechanisms of the TyG index in T2DM patients with albuminuria and CKD.

**Comment 14**: Again, I recommend a native English-speaking revision.

**Response**: Thanks for your suggestion, we have invited a native English-speaking revise the article.

**Comment 15**: For example, in the first paragraph there should be a brief mention of the main results of the study. “The homeostasis model assessment of insulin resistance (HOMA-IR) is a widely used index of insulin resistance in clinical practice. However, due to the need for clinical blood collection and the existence of low utility, this index has certain limitations, and many other simplified clinical indicators can be comparable to it, such as the TG/HDL-C ratio, lipid accumulation index, visceral adiposity index, METS-IR, and TyG index. It was shown that the TyG index can be an indicator with good agreement with the HOMA-IR and that it is a more convenient and quicker way of assessing glycolipid metabolism than the HOMA-IR” The claim that the TyG index does not require blood collection for its measurement, while HOMA-IR does, is incorrect. Both the TyG index and HOMA-IR require blood collection for their measurements. The TyG index is calculated using fasting triglycerides and fasting plasma glucose, both of which necessitate blood collection. Similarly, HOMA-IR, which assesses insulin resistance, also requires blood collection to measure insulin levels and glucose levels.

**Response:** Thanks for your suggestion. We have already described the results of this study in the first section, and I apologize for my incorrect statement. Indeed, both the TyG index and HOMA-IR require blood sampling. Although the TyG index requires a blood sample, it is based on FPG (fasting plasma glucose) and TG (triglycerides), which are readily available and simple to obtain in a clinical setting. However, the HOMA-IR index requires the measurement of serum insulin, which is not routinely tested for in clinical practice and may not be feasible to measure in some remote locations. Therefore, the TyG index is a simple, cost-effective, and easily accessible measure for assessing insulin resistance, so we use this indicator to assess IR.

**Comment 16:** After careful evaluation, I regret to inform you that I cannot recommend this article for publication. In my assessment, the manuscript falls short of meeting even the most basic standards expected of a quality manuscript. Several critical issues have been identified across various sections, including but not limited to, inadequate explanation of the objective and significance of the study in the abstract, lack of clarity and coherence in the introduction, methodological deficiencies, insufficient presentation and interpretation of results, and a discussion lacking in relevance and scientific rigor. Given the substantial shortcomings observed throughout the manuscript, I believe that publishing it in its current form would not contribute positively to the scientific literature.

**Response**: Thanks for your kind suggestions. I'm sorry to say that there are many problems, we have read your suggestions carefully, made general changes to the article and asked native English speaking professionals to make changes to the article, we hope that our changes can make you have a pleasant reading experience, thank you again for your meaningful remarks!

Revised portions are marked in blue in the revised paper. The main corrections in the paper and the responses to the reviewer’s comments are as flowing.

Reviewer #2

**Comment 1**: Li et al. have performed a study on the association between the TyG index and CKD in patients with T2DM. The manuscript is well-written.

Response: Thank you very much for your appreciation and your kind work.

**Comment 2**: The authors have mentioned one limitation of the manuscript. Weren’t there any other limitations in terms of methodology, drafting, and interpreting the results?

Response: We are very grateful to the reviewers for their comments, we couldn't agree with you more, and as you say, there are a few limitations to our research, we made changes, the exact description of which can be seen in the revised manuscript marked in blue and below:

Our study had several limitations. First, it was retrospective, precluding the establishment of a causal relationship between our variables of interest and outcomes, and there may have been reporting and selection biases. Second, although potential confounders were controlled as covariates in the multivariate regression model, it is important to acknowledge that the impact of unrecorded confounders cannot be categorically excluded. Finally, our study was a single-center study with a moderate sample size; future research should include large-scale multicenter longitudinal and prospective studies to further investigate the effects and mechanisms of the TyG index in T2DM patients with albuminuria and CKD.

**Comment 3:** A paragraph discussing the clinical applications of these findings and suggestions for further research is needed in the discussion section. There are several meta-analysis studies that have assessed the TyG index in several diseases (such as HF doi: 10.1186/s12933-023-01973-7, stroke: 10.1186/s12933-022-01732-0, ACS: 10.1186/s12933-023-01906-4, CVDs in the general population: 10.1186/s12933-022-01546-0, AF: 10.1186/s40001-024-01716-8, arterial stiffness: https://doi.org/10.1186/s12933-023-01819-2). These could be discussed in the discussion as among the applications of this novel index.

**Response**: Thank you for your comments and provided the literature, which is very important to me, according to your suggestion, we have added the role and specific application of the TyG index in cardiovascular and cerebrovascular diseases in the revised manuscript, which gives me a deeper understanding of the far-reaching role of TyG index. We have made changes, the exact description of which can be seen in the revised manuscript and below

The TyG index, a new surrogate for insulin resistance measurement, is increasingly supported by evidence of its important role in predicting vascular diseases (41, 42) A meta-analysis indicated that the TyG index is positively associated with the incidence rates of heart failure and adverse outcomes. Similarly, a study also suggests that the prevalence of other common cardiovascular and cerebrovascular diseases, including arteriosclerosis, coronary artery calcification, coronary artery disease, myocardial infarction, and atrial fibrillation, is significantly related to a high TyG index (43-48). However, the association between the TyG index and renal microvascular damage was low. Our study showed that an elevated TyG index is significantly associated with the risk of albumin deficiency and CKD in patients with T2DM. A high TyG index has been shown to exacerbate renal dysfunction by promoting insulin resistance and related metabolic disturbances (49), and it is a reliable and convenient prognostic marker for adverse outcomes in patients with kidney disease (50). A cohort study based on CHARLS data revealed an increased risk of CKD in the highest quartile compared to that in the lowest quartile (51), which is the same as that found in our study. Interestingly, Vinas et al. conducted basic experiments to demonstrate that female mice were more resistant to kidney injury than male mice (52). Saliba et al. reported the same result in humans, which may be related to the estrogen-AMPK pathway (53). However, in our study, we did not find any difference in albumin and CKD levels by sex, even after adjusting for risk factors and sex for stratification; this may be related to the ethnicity of the included population, geographic region, and the number of patients included, which needs to be further explored.

Once again, we would like to thank the editor and all reviewers for their valuable comments and suggestions. Such comments are valuable and helpful for our revision and improvement. We have revised the manuscript accordingly and responded point by point.
