# Supplementary material for: Associations of the TyG index with albuminuria and chronic kidney disease in patients with type 2 diabetes
Source: PLoS One. 2024 Oct 28;19(10):e0312374. doi: 10.1371/journal.pone.0312374 (PMC11515963; doi:10.1371/journal.pone.0312374)
Supplement: Attachment — Submitted filename: Response to Reviewers.docx [file pone.0312374.s006.docx]

**Manuscript ID:** PONE-D-24-07664

**Manuscript title:** Association of TyG index with albuminuria and chronic kidney disease with Type 2 Diabetes Mellitus

Dear Editor:

Thank you very much for your attention, evaluation, and insightful comments on our paper titled "Association of TyG Index with Albuminuria and Chronic Kidney Disease in Type 2 Diabetes Mellitus." We have revised the manuscript in accordance with your valuable advice and the detailed suggestions provided by the reviewers. We have made every effort to address their feedback comprehensively.

We sincerely hope that the revised manuscript will be considered for publication in ***PLOS ONE***. We appreciate the opportunity to resubmit the revised manuscript and are grateful for your time and consideration.

The following is the point-by-point response to the reviewers.

**Reviewer #1:**

**Comment 1:** Is the manuscript technically sound, and do the data support the conclusions? Reviewer #1: No

**Response：**Thank you for your detailed review and valuable feedback on our manuscript. Your comments have been instrumental in improving the quality of our article. We understand your concerns regarding the data source and the conclusion. The data come from the Diabetes Complications Data Set which were collected by the General Hospital of the People's Liberation Army of China from 01/2013 to 12/2017 and it was stored in the Population Health Data Archive. The website is https://www.ncmi.cn/phda/dataDetails.do?id=CSTR:A0006.11.A0005.201905.000282(Language is Chinese). The dataset contains 87 variables for 3,000 patients with T2DM. For our analysis, after passing the inclusion and exclusion criteria, a total of 1048 person was included in the study. The data can be obtained by any approved requester and used for verification. In addition, the General Hospital of the People's Liberation Army of China, located in the capital city of Beijing, is a premier national-level tertiary hospital. It serves a diverse patient population from across the country, which contributes to the reliability and generalizability of our results.

**Comment 2**: Has the statistical analysis been performed appropriately and rigorously?

Reviewer #1: I Don't Know.

**Response:** Thank you very much for your valuable suggestions. We have refined and clarified our data analysis methods as follows:

1) First, we performed normality tests on the data. For normally distributed data, we present results as mean ± standard deviation, while non-normally distributed data are presented as medians (interquartile ranges). Continuous variables were compared using t-tests or Mann-Whitney U tests, and categorical variables were described with frequencies or percentages, with chi-square tests used for inter-group comparisons. These details are included in Table 1 of the revised manuscript.

2) To further investigate the relationship between the TyG index and albuminuria and chronic kidney disease (CKD), we categorized the TyG index into three groups (Q1, Q2, Q3) and performed binary logistic regression analysis. We adjusted for potential confounders such as hypertension, fatty liver, hyperlipidemia, coronary heart disease, total cholesterol, glycosylated hemoglobin, sex, age, fasting glucose, retinopathy, and eGFR. The results demonstrated that the TyG index is an independent risk factor for albuminuria and CKD in T2DM patients. Detailed results can be found in Table 3 and Table 5.

3) Further adjustments for covariates and stratified analyses of variables were performed to validate the results, and the results were consistent with the primary analysis, reinforcing the TyG index as an independent risk factor. Detailed results can be seen in Table 4 and Table 6.

**Comment 3:** Have the authors made all data underlying the findings in their manuscript fully available?Reviewer #1: No

**Response**: Thank you for your patient work, our data comes from publicly available databases. The example data from the Diabetes Complications Data Set can be acquired and was uploaded as S1_Table. Once the application has been approved, all the data can be viewed and used. The website on which the data is obtained is https://www.ncmi.cn/phda/dataDetails.do?id=CSTR:A0006.11.A0005.201905.000282.

**Comment 4**: Is the manuscript presented in an intelligible fashion and written in standard English? Reviewer #1: No.

**Response**: Thank you for your meticulous work. To enhance the quality of the manuscript, we have had it reviewed and edited by a native English speaker to ensure clarity and precision in language.

**Reviewer #2:**

**Comment 1:** Have the authors made all data underlying the findings in their manuscript fully available?Reviewer #2: No

**Response:** Thank you for your advice, our data comes from publicly available databases. The example data from the Diabetes Complications Data Set can be acquired and was uploaded as S1_Table. Once the application has been approved, all the data can be viewed and used. The website on which the data is obtained is https://www.ncmi.cn/phda/dataDetails.do?id=CSTR:A0006.11.A0005.201905.000282.

**Comment 2:** The authors have addressed all the comments by this reviewer and the manuscript is acceptable in its current form.

**Response:** Thanks for your extremely patient work. I couldn't have improved my dissertation without your very instructive advice, thank you for your work on my paper and I wish you all the best.

**Reviewer #3:**

**Comment** 1**:** Li and Wang examined the connection between the TyG index, albuminuria, and CKD in individuals with T2DM. Results indicated a positive association between the TyG index and both albuminuria and CKD, with higher odds ratios observed for those in the highest TyG index quartile. The research topic is interesting and valuable. The revised version addresses all the problems in the first version of the manuscript; the language fluency and punctuation are proper, the article's structure is good, and the discussion section now has an appropriate elaboration of the findings in the context of relative literature. Language and Writing:1. The revised manuscript has proper English fluency and punctuation. The introduction is technically firm and informative.

**Response**: Thank you for your acknowledgement of my paper. I really can't improve my paper without your valuable comments and thank you for the time you have given to my paper.

**Comment 2:** Method:1. The inclusion and exclusion criteria are missing.

**Response:** Thank you for the reminder, I apologize for such an error and I added the inclusion and exclusion criteria. And it is identified in revised the manuscript by the application of red font (line 88-92).

**Comment 3:** The eGFR formula is mistyped. (Line 104)

**Response:** Thank you for your suggestions. I have made the necessary revision to the formula and highlighted the changes in red in revised the manuscript(line108-109).

**Comment 4:** The results is technically firm and informative. The discussion now has an appropriate elaboration of the findings in the context of relative literature and other studies.

**Response:** Thank you for your patient work, I appreciate your suggestive tips to improve my paper.

Once again, we would like to thank the editor and all reviewers for their valuable comments and suggestions. Such comments are valuable and helpful for our revision and improvement. We sincerely hope that these revisions meet your expectations and that the manuscript can be considered for publication in ***PLOS ONE*.** Should you have any further questions or require additional modifications, please do not hesitate to contact us. We are committed to making any necessary revisions and improvements.
